# The Impact of COVID-19 on Patients With Neuromyelitis Optica Spectrum Disorder Beyond Infection Risk

**DOI:** 10.3389/fneur.2021.657037

**Published:** 2021-03-22

**Authors:** Hexiang Yin, Yao Zhang, Yan Xu, Bin Peng, Liying Cui, Shuyang Zhang

**Affiliations:** ^1^Department of Neurology, Peking Union Medical College Hospital, Peking Union Medical College, Chinese Academy of Medical Sciences, Beijing, China; ^2^Neurosciences Center, Chinese Academy of Medical Sciences, Beijing, China; ^3^Department of Cardiology, Peking Union Medical College Hospital, Peking Union Medical College, Chinese Academy of Medical Sciences, Beijing, China; ^4^National Rare Diseases Registry System of China, Beijing, China

**Keywords:** COVID-19, NMOSD, relapse, infection, immunosuppressive therapy

## Abstract

There is an increasing need for better understanding of the impact of coronavirus disease 2019 (COVID-19) on patients with neuromyelitis optica spectrum disorder (NMOSD). A few pilot studies have investigated COVID-19 infections in NMOSD, but few studies have addressed disease activity and immune status of these patients during the pandemic. We carried out a cross-sectional study to examine immune status, relapses, and COVID-19 infections in a cohort of NMOSD patients using an electronic patient registry (MSNMOBase) for multiple sclerosis and related disorders. An online questionnaire was administered to all NMOSD patients in the registry from January 1, 2011, to June 1, 2020. Clinical demographic characteristics, immune status, relapses, treatments, COVID-19 infections, and preventive measures were evaluated. Of the 752 registered patients, 535 (71.1%) with qualified data were included. A total of 486 used preventive therapies during the pandemic, including mycophenolate mofetil (71.2%), azathioprine (13.3%), and other immunosuppressants (6.4%). Neither median immune cell counts nor immunoglobulin levels (*p* > 0.05) were significantly different between patients with or without immunosuppression. During the pandemic, no patients were diagnosed with COVID-19, and the majority (>95%) took one or more effective protective measures (e.g., wearing a mask and social distancing). However, a significantly higher annualized relapse rate (ARR) was observed in the 33 patients with treatment interruptions due to the pandemic compared to before it (*p* < 0.05), whereas ARR changes were not found in patients with continuous treatments or those without treatments (*p* > 0.05). Interruption frequency was significantly higher in patients with relapses compared to those without (34.9 vs. 15.7%, *p* < 0.01). For stable NMOSD patients during the pandemic, the risk of relapse due to treatment interruption may be higher than the risk of COVID-19 infection when protective measures are used, and continuous relapse-prevention treatments may be necessary.

## Introduction

Ever since the emergence of severe acute respiratory syndrome coronavirus-2 (SARS-CoV-2) in December 2019, the virus has rapidly spread and caused the global coronavirus disease 2019 (COVID-19) pandemic (1–4). This pandemic has also raised health concerns for patients with neuromyelitis optica spectrum disorder (NMOSD), a chronic, relapsing autoimmune disorder of the central nervous system mainly associated with antibodies against aquaporin-4 (AQP4) water channels (5, 6). Recently, a subset of NMOSD patients with seronegative AQP4–immunoglobulin G (AQP4-IgG) was found to have serum myelin oligodendrocyte glycoprotein (MOG)–IgG by a cell-based assay (CBA) (7, 8). They were considered to have a novel distinct disease entity, called MOG-IgG–associated disease, as there is increasing evidence suggesting that they have a different pathogenesis and prognosis from patients with seropositive AQP4-IgG (9, 10). An attack of NMOSD commonly results in paralysis and occasionally bulbar dysfunction, which may increase COVID-19 susceptibility and severity by limiting mobility and impairing coughing (5, 6, 11). In addition, the immunosuppressive therapy used for NMOSD relapse prevention can also make these patients potential targets for infections by altering their immune systems. Therefore, COVID-19 infection and NMOSD immunosuppressive therapy in these patients during the pandemic have been the focus of previous several studies (12–15). However, in addition to infection risk, COVID-19 has also influenced other aspects of these patients' lives, including their challenges related to continuous treatment, changes to medical procedures, and mental stresses resulting from the pandemic. These additional factors may increase NMOSD disease activity, with a profound impact on patient quality of life, although to date there are limited data to support this.

We therefore carried out a cross-sectional cohort study on the impact of COVID-19 on NMOSD patients beyond infection risk, as an indicator to clinicians that both infection and relapse risks are important for NMOSD patients during this pandemic era, and to foster better management and outcomes for these patients.

## Methods

### Study Design and Population

Beginning on June 1, 2020, we conducted an online survey using questionnaires mainly focusing on relapses, treatments, behavioral changes, and COVID-19 infections during the pandemic (from December 15, 2019, to June 1, 2020) in NMOSD patients registered in the MSNMOBase, a hospital-based electronic registry for multiple sclerosis (MS) and related disorders established in 2011 (16–18). Additional information was also obtained from patient hospital visits, online visits, and phone calls. From January 1, 2011, to June 1, 2020, a total of 752 NMOSD patients were registered continuously at the time of their diagnoses. All patients met the 2015 International Panel for NMOSD Diagnosis criteria (5) and were seronegative for MOG-IgG. They were followed up until the censoring date. Relapse was defined as the appearance of new neurological dysfunction symptoms, or worsening of existing symptoms, lasting more than 24 h. Relapses were confirmed by well-trained clinicians via phone calls if patients self-reported novel relapses during the pandemic. After excluding patients who did not provide feedback (*n* = 188), patients with insufficient information (*n* = 27), and patients who were outside of China during the pandemic (*n* = 2), 535 (71.1%) NMOSD patients with qualified data were included in the final statistical analysis.

### Data Source

Data were extracted from the MSNMOBase on June 1, 2020. This database holds anonymized and routinely collected (annually or semiannually), longitudinal medical records from patient sources throughout China. Each patient's demographic information, dates of disease onset and all relapses, symptoms, Expanded Disability Status Scale (EDSS) score (19), serum AQP4/MOG-IgG status detected by CBA, complete blood counts, T/B lymphocyte subsets, immunoglobulin level, dates of maintenance treatment initiations, and dates and reasons for drug alterations or terminations were collected.

### Statistical Analysis

Descriptive variables are presented either as a percentage (%) for categorical data or mean ± standard deviation (SD) and median with interquartile range (IQR)/range for continuous data. Differences between the groups were analyzed as appropriate, using χ^2^ tests for categorical data, independent-samples Mann-Whitney *U*-tests, and Wilcoxon matched-pair signed-rank tests for continuous data. No adjustment for multiple comparisons was made. All *p* values were derived from two-sided tests, and the results were considered statistically significant at *p* < 0.05. All analyses were performed using the SPSS software, version 26.0 (SPSS Inc., Chicago, IL, USA).

## Results

### Baseline Patient Characteristics

**Figure 1:**
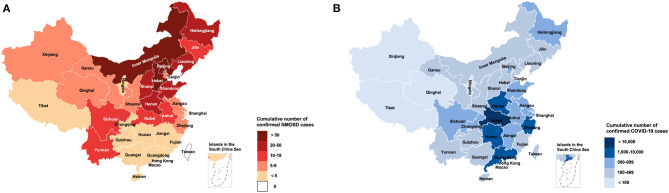
Distribution map of NMOSD cases **(A)** and COVID-19 cases **(B)** across China.

The 535 patients with NMOSD originated from 22 provinces, five autonomous regions, and three municipalities, covering almost all of China. The distribution of the NMOSD cases is presented in Figure 1A, with 12 participants staying in Hubei during the pandemic.

Table 1 summarizes the demographic and clinical characteristics of the subjects. The mean (SD) subject age was 43.8 (14.3) years during the pandemic, with 63 (11.8%) patients older than 60 years. A total of 471 patients (88.0%) were female, and the median (IQR) disease duration was 5.5 (3.2–9.1) years. The median (range) EDSS score at last follow-up was 1.5 (0–8.5), and 462 (86.4%) patients had AQP4-IgG seropositivity. In total, 486 patients (90.8%) received immunosuppressants during the pandemic, including mycophenolate mofetil (MMF, *n* = 381), azathioprine (AZA, *n* = 71), or other immunosuppressants (e.g., hydroxychloroquine, cyclosporine, cyclophosphamide, tacrolimus, methotrexate, *Tripterygium wilfordii* Hook F, and oral glucocorticoids, *n* = 34). The median (IQR) treatment duration was 2.8 (1.7–4.1) years. In total, 49 patients (9.2%) received no treatment. During the pandemic, only 76 patients (14.2%) continued to work outside or attend school, whereas 329 (61.5%) did not work, and 130 (24.3%) worked or studied at home instead. Only one patient had contact with COVID-19 cases; all the others did not. The majority of patients adopted effective protective measures, including wearing a mask (96.4%), reduced social activities (95.9%), hand hygiene (89.7%), indoor air circulation (87.7%), and a healthy lifestyle (73.6%) for preventing infections, with only 0.4% of the subjects reporting no special protection and 9.3% using medications for prevention.

**Table 1:** Demographic and clinical characteristics of patients.

**Characteristics**	
No. of patients	535
Age, mean (SD), years	43.8 (14.3)
Age ≥60 years old, no. (%)	63 (11.8)
Sex ratio, female:male	471:64
Disease duration, median (IQR), years	5.5 (3.2, 9.1)
EDSS score at last follow-up, median (range)	1.5 (0, 8.5)
AQP4-IgG seropositivity, no. (%)	462 (86.4)
Treatment during the pandemic, no. (%)	
Mycophenolate mofetil	381 (71.2)
Azathioprine	71 (13.3)
Other immunosuppressants ^a^	34 (6.3)
None	49 (9.2)
Treatment duration, median (IQR), years	2.8 (1.7, 4.1)
Work status during the pandemic, no. (%)	
No work	329 (61.5)
Work or study at home	130 (24.3)
Work outside or study at school as usual	76 (14.2)
Measures for prevention of COVID-19, no. (%)	
Decrease unnecessary outdoor activities	513 (95.9)
Wear a mask when going out	516 (96.4)
Hand hygiene and disinfection	480 (89.7)
Indoor air circulation	469 (87.7)
Keep healthy lifestyle	394 (73.6)
No special protection required	2 (0.4)
Preventive use of medication	50 (9.3)

a *Hydroxychloroquine (13), cyclosporine (1), cyclophosphamide (8), tacrolimus (3), methotrexate (1), Tripterygium wilfordii Hook F (1), oral steroids (7)*.

### Immune Status During the Pandemic and COVID-19 Infection

**Figure 2:**
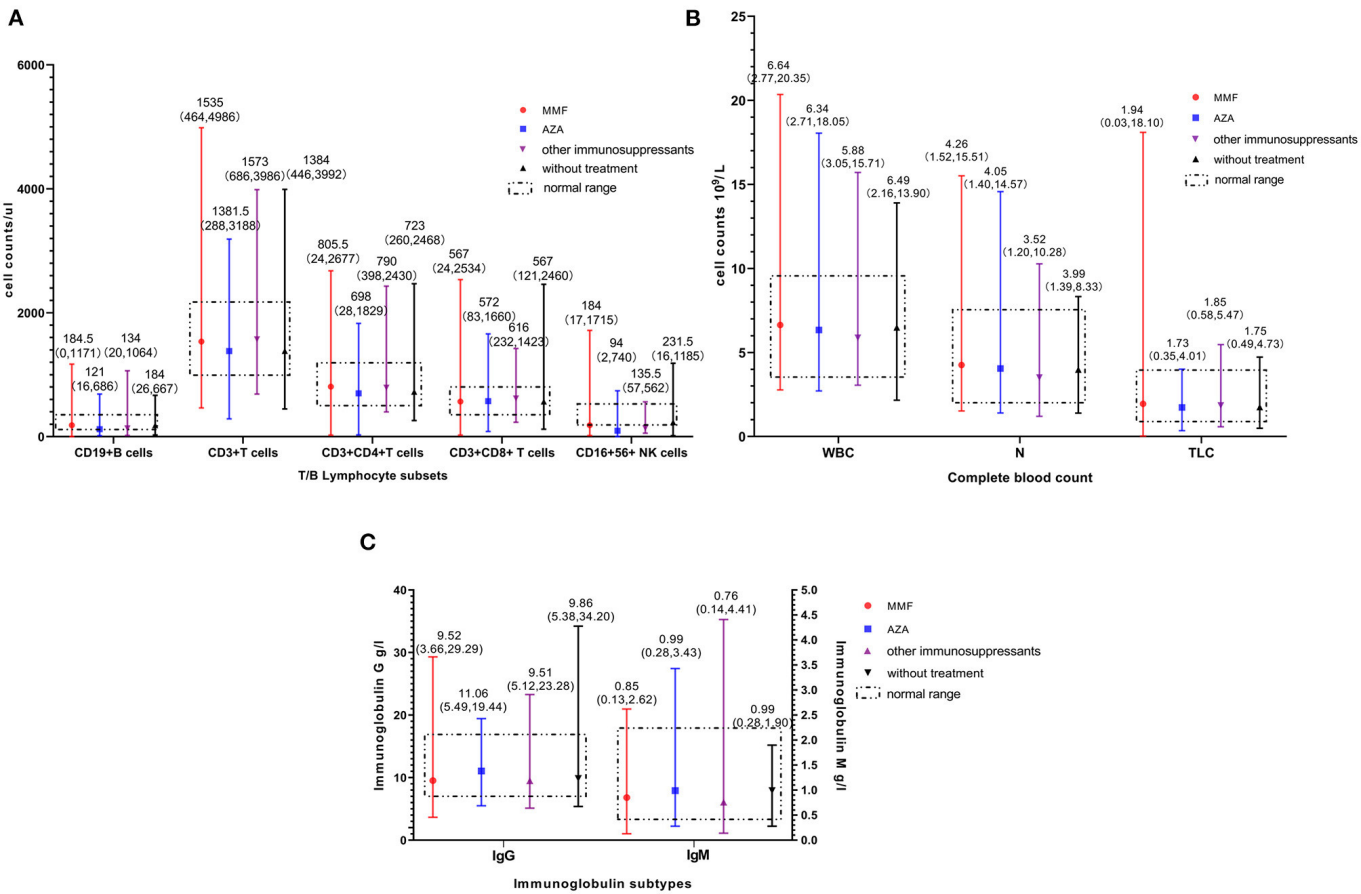
Immune cell count and immunoglobulin level in patients. **(A)** Distribution of T/B lymphocyte subsets. **(B)** Distribution of complete blood count. **(C)** Distribution of different immunoglobulin subtype. Numbers on each vertical line indicate the median (range) value. MMF, mycophenolate mofetil; AZA, azathioprine; WBC, white blood cell; N, neutrophil; TLC, lymphocyte.

Complete blood cell counts, lymphocyte subsets, and immunoglobulin levels (within 6 months) were recorded for 451, 430, and 425 patients, respectively. The detailed median (range) levels for the different immune cells and immunoglobulins in patients without immunosuppressants and those exposed to MMF, AZA, and other immunosuppressants are shown in Figure 2. As presented in Table 2, there were no significant differences between patients with and without immunosuppressants for both median immune cell counts and median immunoglobulin levels.

**Table 2:** Immune status of patients with and without immunosuppressants during the pandemic.

**Immune cell count and immunoglobulin level**	**With Immuno- suppressants**	**Without Immuno- suppressants**	***P* value**
Neutrophil, median (range), ×10^9^/L	4.22 (1.20, 15.51)	3.98 (1.39, 8.33)	0.674
Lymphocyte, median (range), ×10^9^/L	1.88 (0.03, 18.10)	1.75 (0.49, 4.73)	0.968
CD19^+^ B cell, median (range), /μL	171.5 (0, 1,171)	184 (26, 667)	0.445
CD3^+^CD4^+^ T cell, median (range), /μL	787.5 (24.1, 2677.0)	723 (260, 2,468)	0.976
CD3^+^CD8^+^ T cell, median (range), /μL	571.5 (24.3, 2534.0)	567 (121, 2,460)	0.986
Immunoglobulin G, median (range), g/L	9.82 (3.66, 29.29)	9.86 (5.38, 34.20)	0.395
Immunoglobulin M, median (range), g/L	0.87 (0.13, 4.41)	0.99 (0.28, 1.90)	0.110

As of June 1, 2020, a total of 84,570 confirmed cases of COVID-19 were reported in China (National electronic government platform: http://gjzwfw.www.gov.cn/index.html), with the highest level (68,135) of regional infections in Hubei and lowest (1) in Tibet. The distribution of confirmed COVID-19 cases in China is presented in Figure 1B. None of this study's NMOSD patients were diagnosed with COVID-19, or suspected to have it, irrespective of whether they received immunosuppressants, were older than 60 years, or originated from high-risk areas such as Hubei province.

### The Impact of COVID-19 Beyond Infection

The pandemic had an extensive influence on patients' regular follow-ups and treatments. During the pandemic, 33 patients (6.2%) stopped treatments by themselves for fear of infection, only 29% of patients had follow-ups as originally planned, 25% stopped or changed their rehabilitation plans, and 15% reported anxiety during the pandemic.

### Disease Activity During the Pandemic

**Figure 3:**
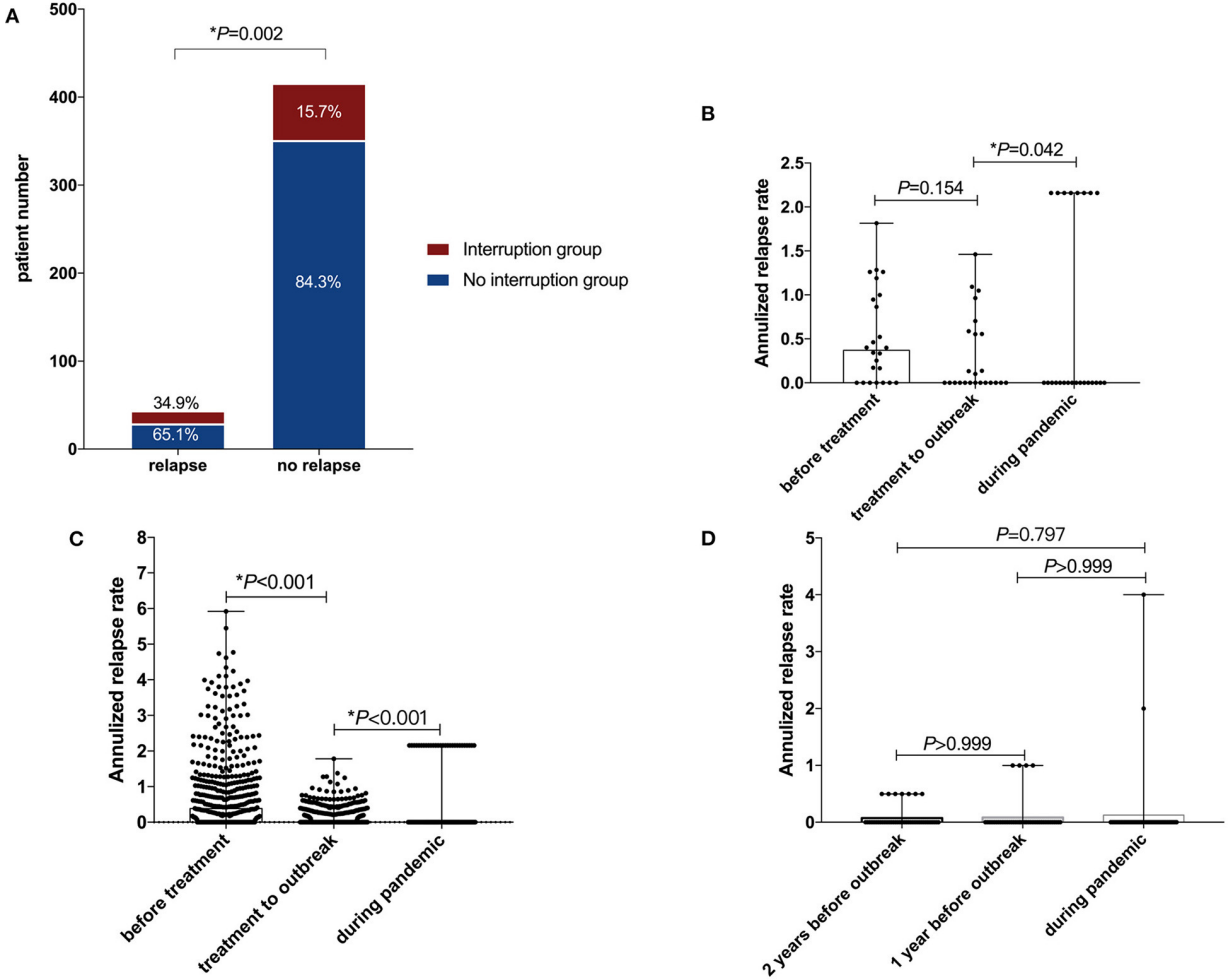
Disease activity during the pandemic. **(A)** Disruption of treatment or follow-up in patients with or without relapse during the pandemic. **(B)** Relapse of patients with treatment disruption. **(C)** Relapse of patients without treatment disruption. **(D)** Relapse of patients without treatment. *p < 0.05 (with significance).

During the pandemic, 44 (8.2%) patients had relapses. Compared with patients without relapses, the percentage of patients who stopped treatment or postponed follow-up in the patients who relapsed was significantly higher (34.9 vs. 15.7%, *p* < 0.01) (Figure 3A).

Additionally, for 33 patients with treatment interruption, compared with the annualized relapse rate (ARR) before treatment, the ARR after treatment but before the pandemic decreased with a median (IQR) difference of −0.083 (−0.765, 0.196) (*p* = 0.147). However, the intrapandemic ARR increased significantly, with a median (IQR) difference of 0 (−0.137, 1.713) (*p* = 0.042) (Figure 3B). For the 453 patients with continuous treatments during the pandemic, the ARR after treatment (but before the pandemic) decreased significantly compared to the ARR before treatment, with a median (IQR) difference of −0.276 (−0.982, 0) (*p* < 0.001), and the ARR even decreased significantly during the pandemic, with a median (IQR) difference of 0 (−0.238, 0) (*p* < 0.001) (Figure 3C). For the 49 patients without immunosuppressant treatment, there were no significant ARR differences during the pandemic, or one or 2 years before the pandemic (*p* > 0.05) (Figure 3D).

## Discussion

To date, COVID-19 has created crises in every aspect of patients' lives. Compared to smaller previous studies, the present study assessed the risks of infection, disease activity, and immune status in a large sample size of NMOSD patients during the COVID-19 pandemic. We found no significant differences in immune status between patients using, and not using, immunosuppressive therapies. No NMOSD patients in this study were diagnosed with COVID-19, and a majority (>95%) took one or more effective protective measures (e.g., wearing a mask and social distancing), but patients with treatment disruptions experienced significantly more relapses during the pandemic.

The immune response against COVID-19 begins with the innate immune system (e.g., natural killer cells, macrophages, and cytokines) and then shifts to an adaptive immune response mediated mainly by T cells. Any long-term immunity against the virus is based on a humoral response mediated by B cells (20–23). Therefore, patients using therapies that target T/B cells are considered to be at higher risk of infections, including COVID-19. Recently, efficacy of B cell-depleting therapies has been proven in both NMOSD and MS patients. The current data about the impact of these medications on COVID-19 are mainly from MS patients and the results are inconsistent. Some early pharmacovigilance analysis and case reports showed that B cell-depleting agents did not increase the severity of COVID-19, whereas a recent large-scale study of 200 MS patients with treatment using an anti-CD20 monoclonal antibody noted a higher infection frequency of COVID-19 and a more severe course (24, 25). Besides the B cell-depleting agents, MMF and AZA, maintenance therapies for the majority of our subjects, have also been proven to be effective in preventing NMOSD relapses in several studies (26–29), and both therapies were shown to have antiproliferative effects on T/B cells through the purine-synthesis interference. Here, although some patients being treated with immunosuppressives (especially MMF and AZA) had lower immune cell counts and immunoglobulin levels than normal, these lower levels were similar to those of patients without treatment. With the preventive measures suggested by the World Health Organization (3), none of these abnormal immune status patients had COVID-19 infections, even patients from the high-risk Hubei area, confirming similar reports (13, 15, 30) that the risk of COVID-19 infection did not increase in NMOSD patients being treated with immunosuppressants. In fact, infection risk is an ongoing concern for all patients using immunosuppressive therapies even without the COVID-19 pandemic, and our data further support the safe use of immunosuppressive therapies in conjunction with effective preventive approaches in future pandemic scenarios.

In contrast to a relatively low infection risk, a significant increase in disease activity was observed during the pandemic in our cohort of NMOSD patients. Stressful events have been reported to exacerbate MS by inducing immunological changes (31–35). The COVID-19 pandemic has also brought increased stress to the general population (36), and a pilot study involving 33 patients with either NMOSD, MS, or another related disorder revealed that two NMOSD patients experienced relapses attributed to stress, but none had COVID-19 infections during the pandemic (37). However, in our study, only patients with interrupted treatments had significantly increased ARRs during the pandemic, although ARR differences before and after treatments did not reach statistical significance possibly because of an insufficient number of patients and the heterogeneity of their treatments. In contrast, patients with continuous treatments and those without treatment did not demonstrate similar ARR changes. In addition, the percentage of relapse patients with treatment and follow-up interruptions was significantly higher compared to patients without relapses. These results highlight the importance of ongoing treatments for NMOSD patients during the pandemic. Compared to injected medications, oral agents such as MMF and AZA may have an advantage for increasing treatment compliance during the pandemic as patients wish to avoid frequent hospital visits.

Several vaccines against COVID-19 (e.g., viral protein and nucleic acid vaccines, artificial antigen-presenting cell vaccines, surrogate viral vector vaccines, and live-attenuated vaccines) are being developed (23, 38–40). Although we found that NMOSD patients using immunosuppressive therapies did not have a higher COVID-19 risk and that ongoing treatments during the pandemic were recommended considering possible increased disease activity, some immunotherapies may impact future COVID-19 vaccines. A previous study found that patients using B cell depletion therapies had negative SARS-CoV-2 serology after confirmed COVID-19 infections (41). This should prompt clinicians to evaluate the potential impact of specific therapies on infection risk, severity, and immune response to vaccination when making any new clinical decisions. Here, key immune components (e.g., B cells and immunoglobulins) that play vital roles in the immune response to vaccination were not significantly different in patients with and without immunosuppression. This suggests that oral immunosuppressants such as MMF and AZA may have little impact on COVID-19 vaccines, another advantage for their administration during the pandemic. The real impact of different medications on future COVID-19 vaccines will demand more clinical evidence in the future.

In this study, we still included 73 patients with seronegative AQP4-IgG, who might have different pathogenesis, treatment response, and susceptibility to and severity of COVID-19 from those with seropositive AQP4-IgG. However, none of the NMOSD patients were diagnosed with or suspected to have COVID-19 during the pandemic, regardless of whether they were AQP4-IgG seropositive or seronegative. A future larger-scale study is necessary to explore the occurrence of COVID-19 in NMOSD patients with seronegative AQP4-IgG.

This study has several limitations. It is based on single-center data from China, and the majority of participants were from areas with low COVID-19 incidence. In addition, the study was only carried out within 6 months of the COVID-19 outbreak. Further data collection on the impact of the pandemic on NMOSD patients in higher-risk areas, for longer periods of time, will be necessary in the future. The nucleic acid and antibody testing for SARS-CoV-2 was voluntary and was performed mostly in patients with symptoms or who were known to have been in contact with confirmed cases of COVID-19. Therefore, the infection risk of the whole study population may have been underestimated. Nevertheless, our investigation included a large sample size, with wide geographical coverage, and provides evidence for maintaining ongoing immunosuppressive therapies for NMOSD patients during the pandemic.

## Conclusion

The management of NMOSD patients in the pandemic era should balance infection risk, disease activity, and possible vaccination impact. For stable NMOSD patients, the risk of relapse due to treatment interruption may outweigh the risk of COVID-19 infection, and ongoing immunosuppressive therapies are warranted during the pandemic.

## Data Availability Statement

The data that support the findings of this study are available from the corresponding author upon reasonable request.

## Ethics Statement

The institutional review board of Peking Union Medical College Hospital approved this study. Informed consent was not required for the use of the anonymized data set.

## Author Contributions

HY and YZ: study concept and design: acquisition, analysis, or interpretation of data, statistical analysis, and drafting of the manuscript. YX: study concept and design: acquisition, analysis, or interpretation of data: critical revision of the manuscript for important intellectual content: study supervision. BP, LC, and SZ: critical revision of the manuscript for important intellectual content. YX and SZ: obtained funding. All authors contributed to the article and approved the submitted version.

## Conflict of Interest

The authors declare that the research was conducted in the absence of any commercial or financial relationships that could be construed as a potential conflict of interest.
